# Temporal force governs the microbial assembly associated with *Ulva fasciata* (Chlorophyta) from an integrated multi-trophic aquaculture system

**DOI:** 10.3389/fmicb.2023.1223204

**Published:** 2023-10-05

**Authors:** Dzung Nguyen, Ofer Ovadia, Lior Guttman

**Affiliations:** ^1^Marine Biology and Biotechnology Program, Department of Life Sciences, Ben-Gurion University of the Negev, Eilat, Israel; ^2^Israel Oceanographic and Limnological Research, The National Center for Mariculture, Eilat, Israel; ^3^Department of Life Sciences, Ben-Gurion University of the Negev, Be'er Sheva, Israel

**Keywords:** *Ulva fasciata*, microbial assembly, temporal dynamics, succession, IMTA

## Abstract

*Ulva spp*., one of the most important providers of marine ecosystem services, has gained substantial attention lately in both ecological and applicational aspects. It is known that macroalgae and their associated microbial community form an inseparable unit whose intimate relationship can affect the wellbeing of both. Different cultivation systems, such as integrated multi-trophic aquaculture (IMTA), are assumed to impact *Ulva* bacterial community significantly in terms of compositional guilds. However, in such a highly dynamic environment, it is crucial to determine how the community dynamics change over time. In the current study, we characterized the microbiota associated with *Ulva fasciata* grown as a biofilter in an IMTA system in the Gulf of Aqaba (Eilat, Israel) over a developmental period of 5 weeks. The *Ulva*-associated microbial community was identified using the 16S rRNA gene amplicon sequencing technique, and ecological indices were further analyzed. The *Ulva*-associated microbiome revealed a swift change in composition along the temporal succession, with clusters of distinct communities for each timepoint. *Proteobacteria, Bacteroidetes, Planctomycetes*, and *Deinococcus-Thermus*, the most abundant phyla that accounted for up to 95% of all the amplicon sequence variants (ASVs) found, appeared in all weeks. Further analyses highlighted microbial biomarkers representing each timepoint and their characteristics. Finally, the presence of highly abundant species in *Ulva* microbiota yet underestimated in previous research (such as phyla *Deinococcus-Thermus*, families *Saprospiraceae, Thiohalorhabdaceae*, and *Pirellulaceae*) suggests that more attention should be paid to the temporal succession of the assembly of microbes inhabiting macroalgae in aquaculture, in general, and IMTA, in particular. Characterizing bacterial communities associated with *Ulva fasciata* from an IMTA system provided a better understanding of their associated microbial dynamics and revealed this macroalgae's adaptation to such a habitat.

## 1. Introduction

*Ulva fasciata*, a species belonging to the *Ulva* green macroalgae genus, plays a pivotal role in brackish and marine ecosystems as ecosystem engineers (Lobban and Wynne, 1981). Like other marine eukaryotes such as coral (Rosenberg et al., 2007; Bourne et al., 2009) and marine invertebrates (Dubilier et al., 2008), macroalgae as a group of photosynthetic sessile organisms (Florez et al., 2017) also maintain a stable intimate relationship with their microbiota to form a consolidated system (Egan et al., 2013). Associated bacteria can provide macroalgae with vital nutrients and vitamins for normal growth (Provasoli and Pintner, 1980; Croft et al., 2005), contribute to nitrogen fixation (Penhale and Capone, 1981), inhibit undesirable colonies, and detach biofouling species from the seaweed (Dobretsov et al., 2006; Egan et al., 2008; Wiese et al., 2009; Ismail et al., 2016). In macroalgal species such as *Ulva fasciata* (Singh et al., 2011) and others [*Ulva linza, Ulva compressa* (Fries, 1975), and *Ulva pertusa* (Nakanishi et al., 1999)], epiphytic bacteria are known to play a substantial role in their morphological development. From a mutually beneficial perspective, seaweeds are dependent on the associated microbiome to complement their functions while they, in turn, provide essential niches and resources that can assist the microbial community settlement (van der Loos et al., 2019).

Given the importance of the *U. fasciata*-associated microbiota, it is important to note that specific environments and conditions can determine its resident bacteria, leading to a selection of different microbial compositional guilds and directly impacting seaweed physiology (van der Loos et al., 2019). Integrated multi-trophic aquaculture (IMTA) refers to a system where fed and extractive species are grown together to increase the recovery of nutrient residues and hence decrease the environmental footprints of aquaculture activities (Neori et al., 2019). It was suggested that the high abundance of the class *Alphaproteobacteria* in aquaculture habitats may contribute to the growth of *Ulva* (Califano et al., 2020). However, due to current limited research on macroalgae in IMTA systems, further experiment-based studies are required to corroborate this knowledge. In Israel, *Ulva* has been utilized as a crucial part of the IMTA biofiltration system for several decades due to its capability to assimilate excess nutrients (most importantly ammonia) from fish farm effluents (Shpigel et al., 2019; Shahar and Guttman, 2020). A study on macroalgal bacteria (Califano et al., 2020) revealed that an IMTA caused changes in the prokaryotic community associated with *Ulva* compared with that from the wild. Though this was among the important pioneer studies on *Ulva* microbiota in an IMTA setting, there remains a gap in monitoring the microbial community along the temporal dimension under the same culture mode. Temporal dynamics are expected to play a crucial part in shaping the microbial assembly in algal thalli and thereby constitute a stepping stone for more studies regarding macroalgae and microbial symbioses. Generally, very few studies have tried to characterize the *U. fasciata*-associated microbial community, especially in an IMTA environment. Given that *Ulva* spp. have become a universally popular component of the IMTA system, it becomes relevant to study their associated microbiota for ecological and practical purposes.

Based on this background, our research aimed at the characterization of the microbial community associated with *U. fasciata* during a period of 5 weeks under an IMTA setup. The results will not only contribute to the knowledge gap about the taxonomic diversity of the algal microbiome in an IMTA system but also provide insights into the temporal dynamics of this community in specific conditions for further application.

## 2. Materials and methods

### 2.1. Experiment setup

**Figure 1:**
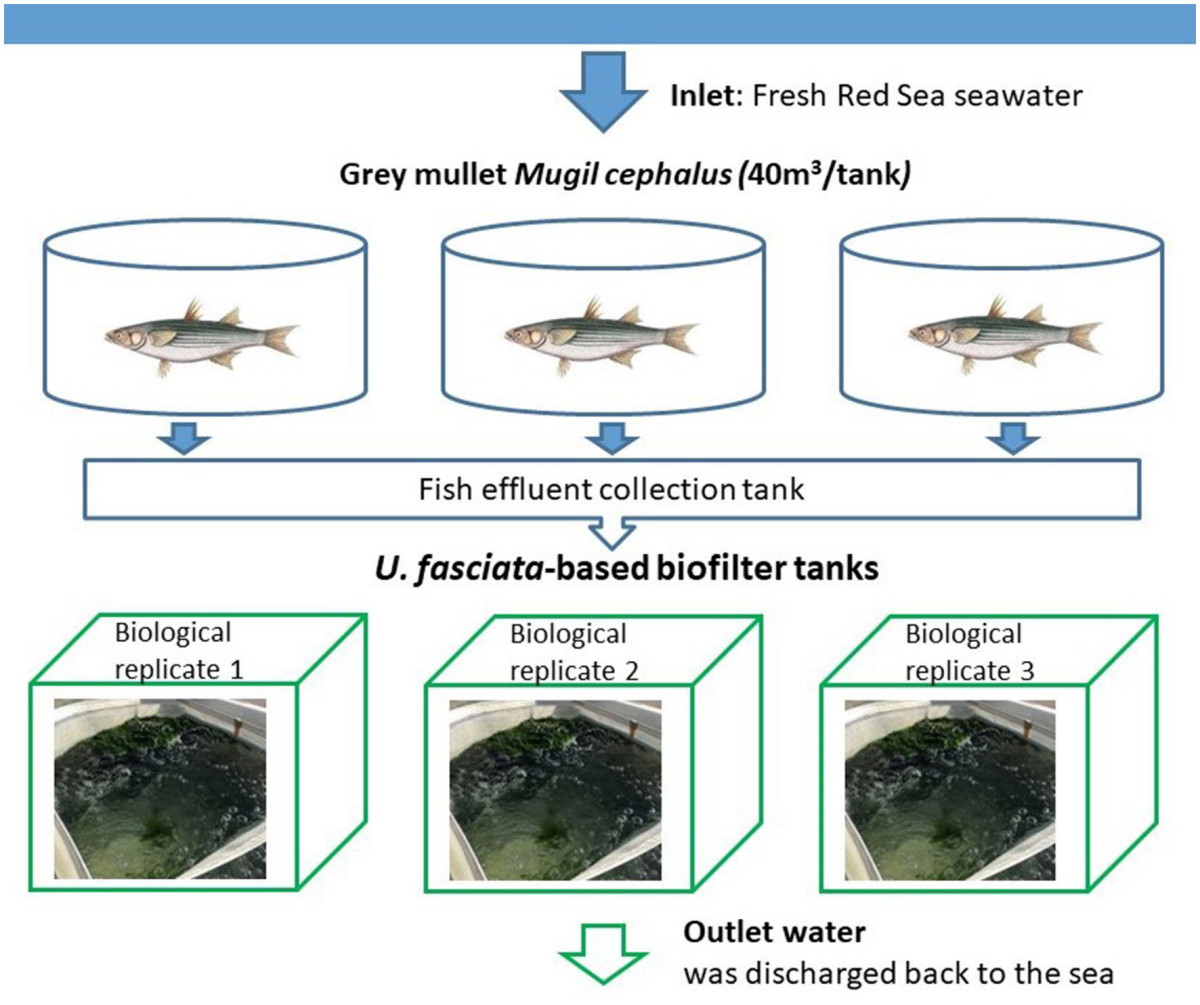
Experimental setup of the *U. fasciata*-based biofilter system. Effluents from fish tanks were routed to the sedimentation pond and enriched with nutrients, then pumped to the biofilter system consisting of three biological replicates.

The experiment took place for 5 weeks in March–April 2020. The system was designed following a previous study where *U. fasciata* was integrated as a biofilter at the Israel Oceanographic and Limnological Research—The National Center for Mariculture (IOLR-NCM) in the Gulf of Aqaba (Eilat, Israel) (Shahar and Guttman, 2020). Seawater was pumped to fish tanks from a location of ~300 m offshore (32°29′N and 58°34′E) from a depth of 13 meters (Nguyen et al., 2023). Following the original concept of IMTA, three fishponds (40 m^3^ each) of gray mullet (*Mugil cephalus*) were located upstream of the *Ulva*-based biofilter (Figure 1), from which the effluent water was fed into the *Ulva* cultivation tanks. The flow rate of input seawater and effluent discharge was ~4 m^3^ d^−1^. Additional nutrients that support the optimal performance of the seaweed were supplied from a 250 L tank through a dosing pump (VMS 2001, EMEC, Italy), resulting in a volume that comprised 10% of the total flow from the *Ulva*'s inlet water source. Nutrient concentrations of the cultivation water were measured during the experimental period, including TAN-N, NO_3_-N, and PO_4_-P with respective inlet concentrations of 1.51 ± 0.47, 0.92 ± 0.76, and 0.19 ± 0.09 mg L^−1^ and outlet concentrations of 1.24 ± 0.70, 0.88 ± 0.69, and 0.18 ± 0.12 mg L^−1^ (Supplementary Table 1A). In addition, additional relevant physical parameters were also measured throughout the experiment, such as temperature (20.84 ± 1.6°C), dissolved oxygen (7.91 ± 0.52 mg/L or 113.33% ± 6.93%), and pH (7.99 ± 0.05) (Supplementary Table 1B). At the beginning of the experiment, 1 kg of fresh *Ulva fasciata* was collected from a production tank at IOLR-NCM and transferred to the experimental tanks holding a volume of 550 L each. An aeration system was installed at the bottom of each tank to control the concentration of oxygen and ensure that algae were kept circulating in the water column (Guttman et al., 2018). The macroalgae were harvested on a weekly basis so that only 1 kg of *U. fasciata* would be retained for development.

### 2.2. *U. fasciata* sample collection

Algal thalli were collected once a week and distributed into different samples as water brings the thalli to the upper part of the tank during aeration. Five technical replicates (~20 g each) consisting of three thalli each were harvested from each tank, stored in sterile plastic bags filled with water from the sample tank, placed in an icebox, and transported to the laboratory (Penesyan et al., 2009; Ismail et al., 2018; Califano et al., 2020). In the laboratory, under sterile conditions, we chose only three healthy samples having green thalli from each tank for further analyses. Sessile organisms on the surface of algal thallus were checked carefully and, if present, were carefully removed with a sterile scraper (Califano et al., 2020). The algal samples were, then, washed three times, filtered, and autoclaved natural seawater to remove loosely associated bacteria (Jiang et al., 1999; Burgess et al., 2003). After the checkup, the samples were stored at −80°C until DNA extraction (Califano et al., 2020).

### 2.3. DNA extraction and amplification

From each sample, an approximate biomass of 200 g of algal thalli was subsampled and cut into smaller pieces for DNA extraction. The algal DNA was extracted using the PureLink^TM^ Microbiome DNA Purification Kit (Thermo Fisher Scientific) following the manufacturer's protocol. The bacterial community was identified using the primer set 515 Fa/926 R, targeting the V4–V5 hypervariable region of the 16S rRNA gene as recommended by the Earth Microbiome Project (Caporaso et al., 2012; Walters et al., 2016). The PCR performance included initial denaturation at 95°C for 5 min, 28 cycles of 94°C for 45 s, 50°C for 60 s, 72°C for 90 s, and final elongation at 72°C for 10 min. PCR products were analyzed via gel electrophoresis in 2.0% agarose and then stored at −20°C until sequencing (https://earthmicrobiome.org/).

### 2.4. Amplicon sequencing and data processing

Sequencing was performed on an Illumina MiSeq at Chicago Research Informatics Core (University of Illinois). The merging of forward and reverse reads was conducted by Paired-End Read Merger (PEAR) (Zhang et al., 2014), after which adapters, primers, and ambiguous nucleotides were discarded from the reads. Chimeric sequences were identified using the USEARCH algorithm (Alloui et al., 2015) as compared with a reference database. Amplicon sequence variants (ASVs) were picked up using deficiency of adenosine deaminase 2 (DADA2) at a 97% similarity threshold, while the taxonomy was annotated via the Naive Bayesian approach under the DADA2 package for bacteria (Callahan et al., 2016). Taxonomy assignment for prokaryotes (bacteria and archaea) was, then, completed by using the Silva database (Quast et al., 2012) as a reference. Five samples that had a very low number of sequences (<150 counts/sample) were discarded from the whole dataset; therefore, in the end, only 40 samples were further analyzed during the 5-week experiment (Supplementary Table 2A). Filtration was performed to exclude chloroplast and mitochondria from the samples, which culminated in an average of 53.98% retained sequences in all samples (Supplementary Table 2B). A final clean-up step was conducted to remove 334 low-count ASVs that were present in <10% of the samples for the downstream analyses.

### 2.5. Statistical analyses

All samples were rarefied at the minimum library size of 10,000 reads. Based on the Shannon index, alpha diversity was calculated for all samples; then, statistical test of Kruskal–Wallis was used to measure the significant difference between the diversity of samples along 5-week timepoints. Beta diversity of the community was determined by the statistical tests of permutational multivariate analysis of variance (PERMANOVA) based on the Bray–Curtis metrics and then subjected to ordination by the principal coordinate analysis (PCoA). Statistical tests were performed in R version 4.2.0 (R Development Core Team, 2010) using the vegan package version 2.6-2 (Oksanen et al., 2022). Ecological indices were calculated, and figures were created using the web-based tool MicrobiomeAnalyst (Chong et al., 2020). The linear discriminant analysis (LDA) effect size (LEfSe) method was utilized to identify time biomarkers—the features (ASVs) that mostly explained the differences between samples originating from different weeks (Segata et al., 2011). In this method, the non-parametric Kruskal–Wallis rank-sum test was first used, followed by the LDA to examine the effect size of the significant features (Chong et al., 2020).

## 3. Results

### 3.1. Composition of the microbial community associated with *U. fasciata*

**Figure 2:**
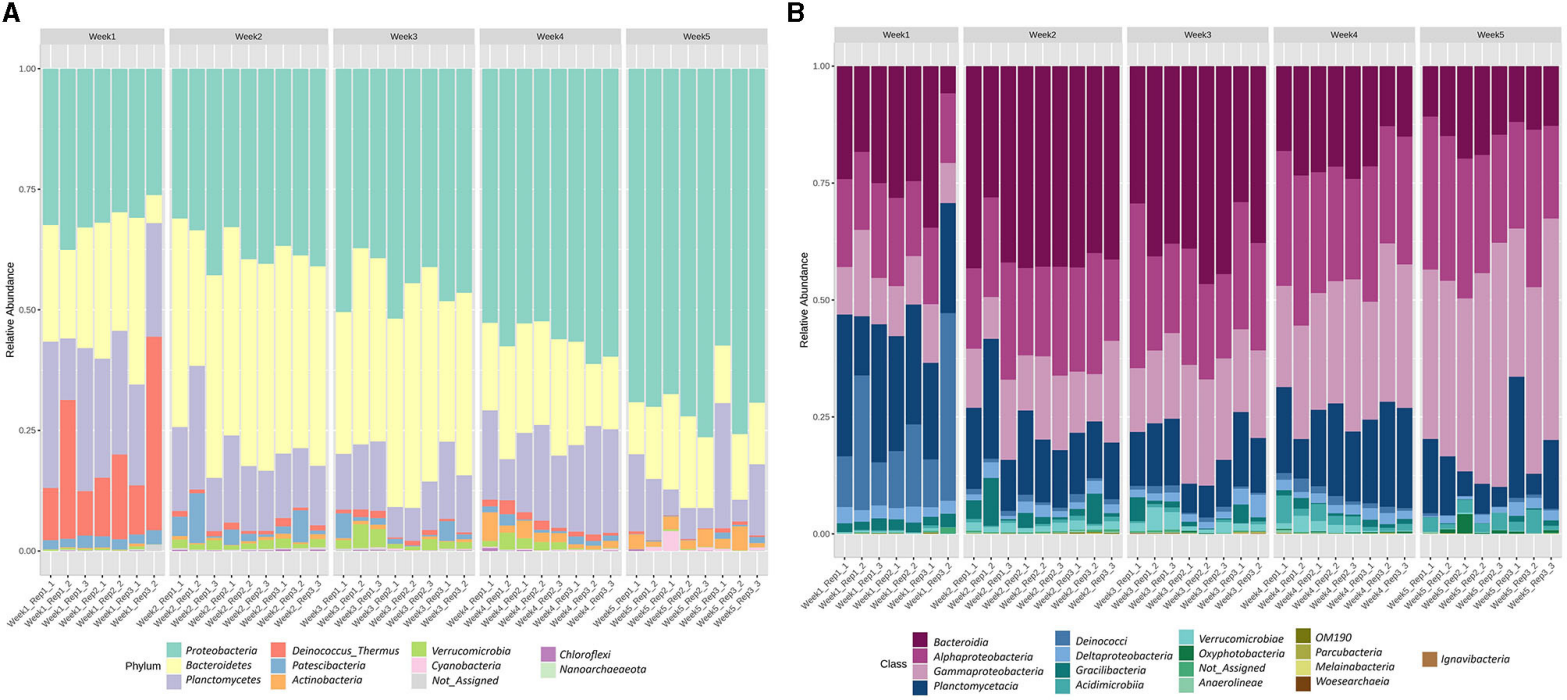
Taxonomic composition performed as the relative abundance of the bacteria associated with *U. fasciata* at **(A)** phylum and **(B)** class levels across 5 weeks of cultivation.

There were 2,421,985 reads of prokaryotes sequenced from the samples, with an average of 60,549 reads per sample. A total of 911 ASVs were identified in the microbial communities associated with *U. fasciata* samples during 5 weeks of maturation, including 11 bacterial phyla. Four of these bacterial phyla accounted for up to >95% of the total sequence abundance in the following decreasing order: *Proteobacteria, Bacteroidetes, Planctomycetes*, and *Deinococcus-Thermus* (Figure 2A and Supplementary Table 3). Bacteria belonging to phylum *Proteobacteria* were enriched along the temporal development of the microbial community on *Ulva*, while *Bacteroidetes* were more abundant during weeks 2 and 3. *Planctomycetes* were consistently present along the 5 weeks while *Deinococcus-Thermus* predominated the initial successional stage. At the class level, the most prevalent classes which were present in over 10% of all samples were *Bacteroidia* (27.16%), *Alphaproteobacteria* (23.24%), *Gammaproteobacteria* (22.75%), and *Planctomycetacia* (15.69%) (Figure 2B and Supplementary Table 4). While *Bacteroidia* showed a decreasing trend toward the last 2 weeks of succession, both *Alphaproteobacteria* and *Gammaproteobacteria* shared the same increasing tendency throughout 5 weeks (Supplementary Table 4). At the family level, the most dominant taxa were *Saprospiraceae* (19.77%), *Thiohalorhabdaceae* (19.32%), and *Pirellulaceae* (13.60%) (Supplementary Figure 1 and Supplementary Table 5), while at the genus level, the most highly annotated taxa included *Granulosicoccus* (19.32%), *Blastopirellula* (11.23%), and *Erythrobacter* (7.28%) (Supplementary Figure 2 and Supplementary Table 6).

### 3.2. Alpha diversity of the microbial community associated with *U. fasciata*

**Figure 3:**
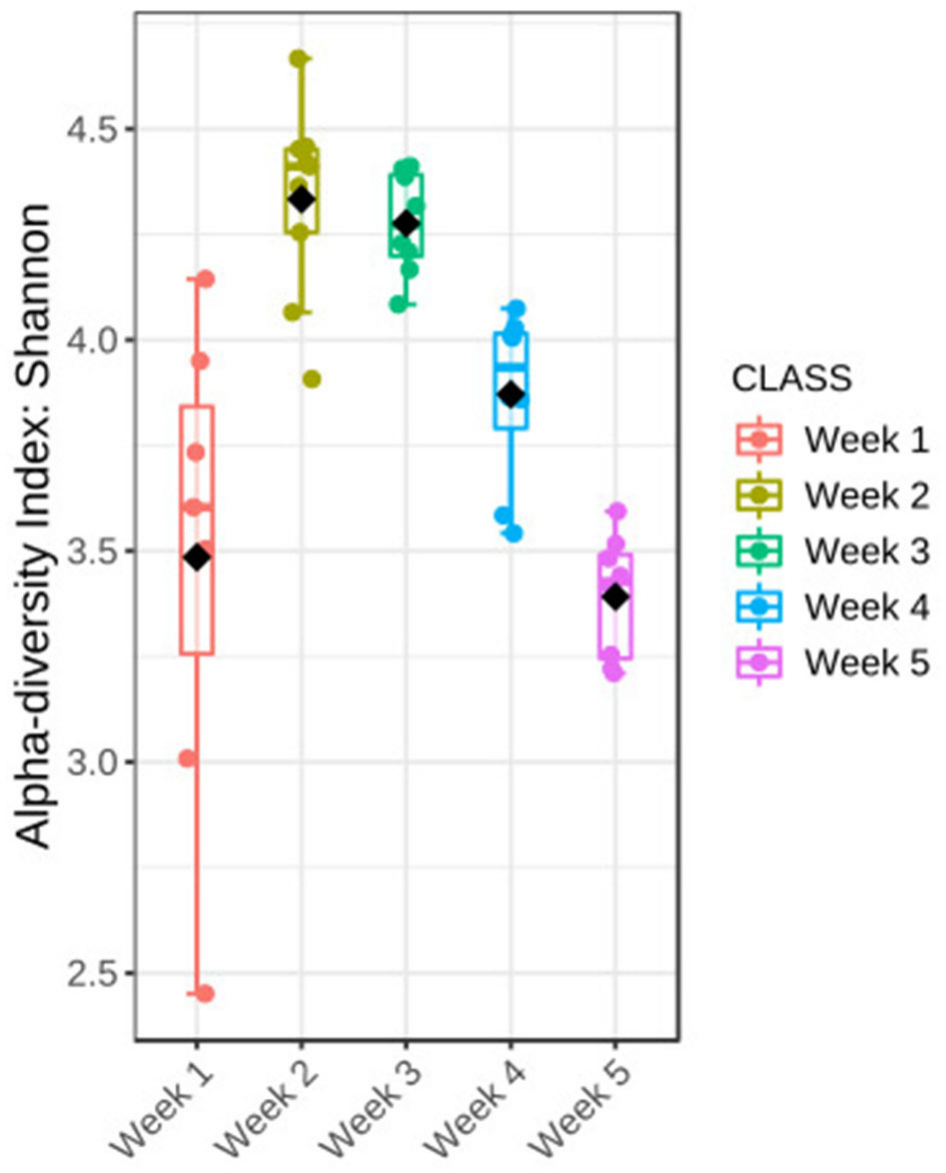
Changes in the bacterial community associated with *Ulva* during 5 weeks of temporal succession were shown by Shannon diversity; statistical Kruskal–Wallis test, *H*-value = 29.63, *p*-value < 0.001. The test was performed at the feature level; box plots show a 95% confidence interval, bar plots represent the standard error, straight lines represent the median, and black dots show the average values, *n* = 40.

Diversity of the microbial community was characterized during a 5-week cultivation period of *Ulva fasciata* under the IMTA setup. The Shannon index was used to measure the diversity of the microbial community associated with *U. fasciata*. It showed considerable changes in the community diversity, with significant differences evident between the weekly samples (Figure 3, Kruskal–Wallis test, H value = 29.63, *p*-value < 0.001). The highest Shannon value was in the second timepoint with an average of 4.3, while the lowest value identified was at the last timepoint with a diversity index of ~3.3. The microbial community revealed the broadest variability in terms of diversity value in the first week compared with other samples from other timepoints.

### 3.3. Beta diversity of the bacterial community

**Figure 4:**
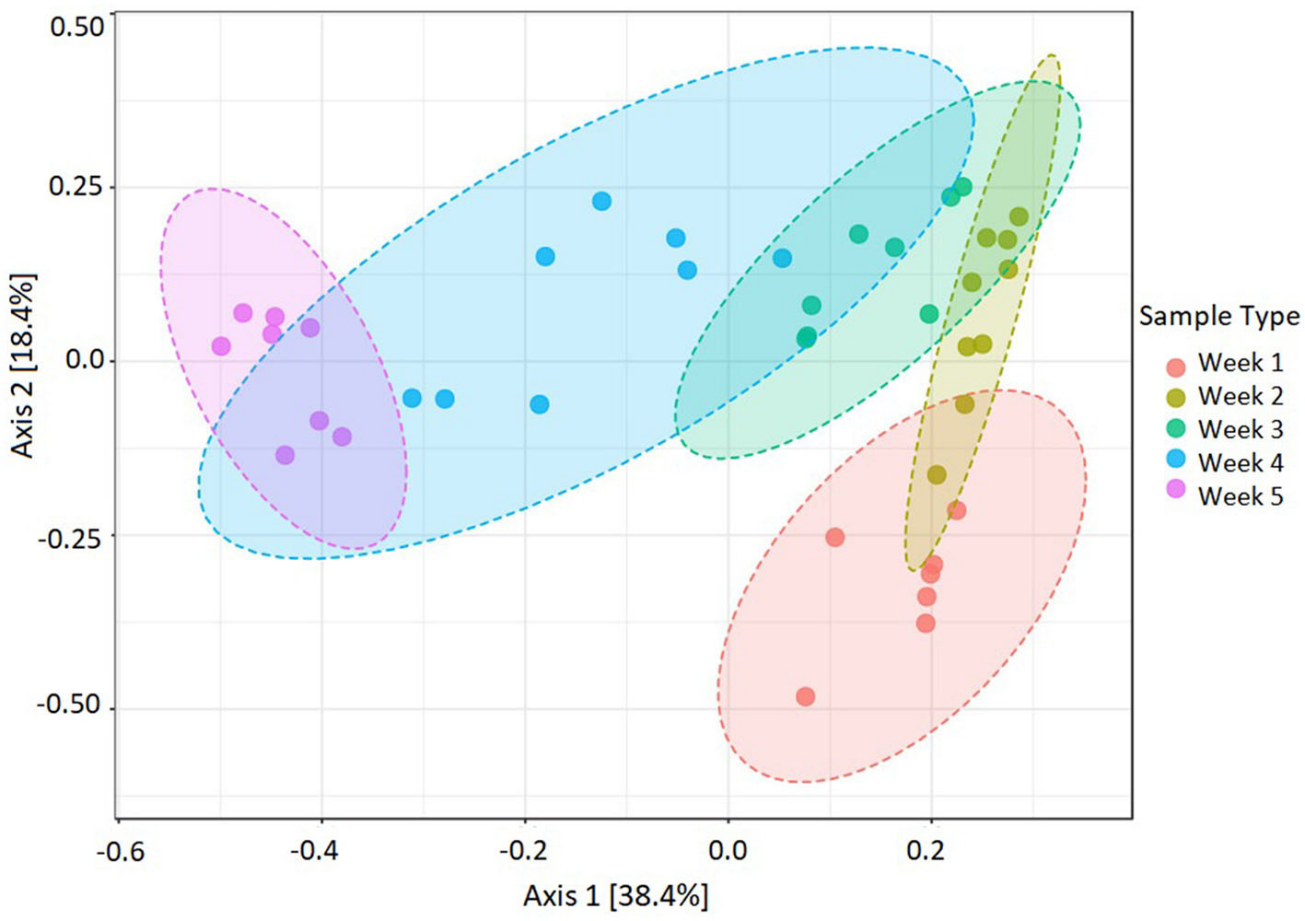
Dissimilarities in *Ulva*-associated microbial community during 5-week assembly performed by beta diversity at the feature level. Statistical test PERMANOVA based on Bray–Curtis metrics, then subjected to ordination on PCoA (*F*-value = 14.12, *R*-squared = 0.62, *p*-value < 0.001, *n* = 40).

**Figure 5:**
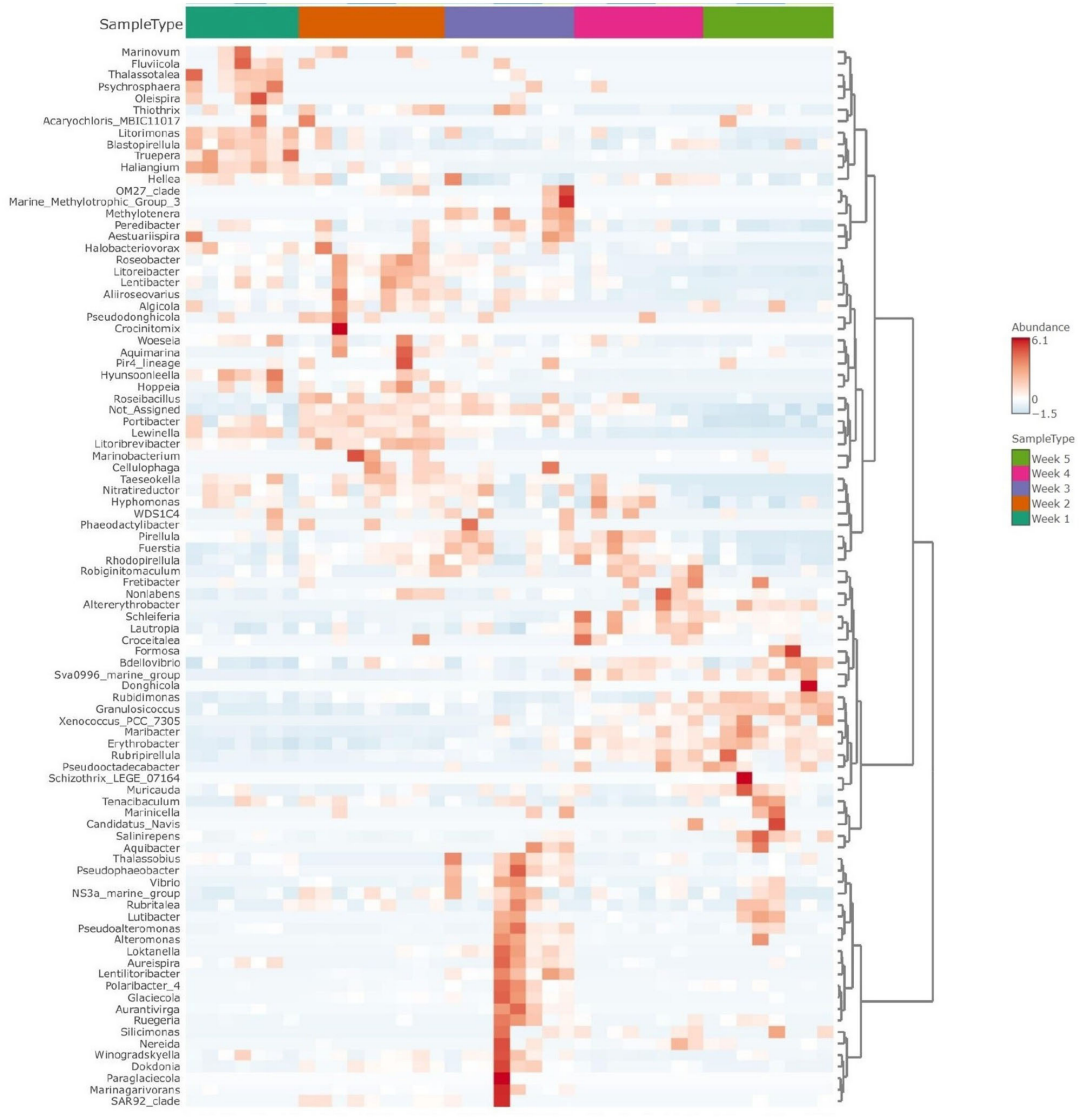
Clustered heatmap shows taxonomic abundance variance following temporal assemblies (weeks 1 to 5) for *Ulva*-associated bacteria community. The graph is shown at the genus level, with red color representing more abundant taxa and blue color indicating the less abundant ones.

The beta diversity between five microbial communities associated with *U. fasciata* was calculated based on Bray–Curtis metrics. The statistical test of PERMANOVA demonstrated significant clusters according to the temporal origin of the samples (Figure 4, *F*-value = 14.12, *R*-squared = 0.62, *p*-value < 0.001). When subjected to PCoA, the first and second axes explained up to 56.8% of the total variation across samples. Along the most explanatory axis, the first- and second-week communities tended to separate the farthest from the remaining communities. This result was evidently supported by the temporal cluster, forming distinct communities along the 5-week succession (Figure 5). As the community swiftly changed along developmental stages, each week was represented by the presence of different predominating taxonomic groups.

### 3.4. Biomarkers of the microbial community

**Figure 6:**
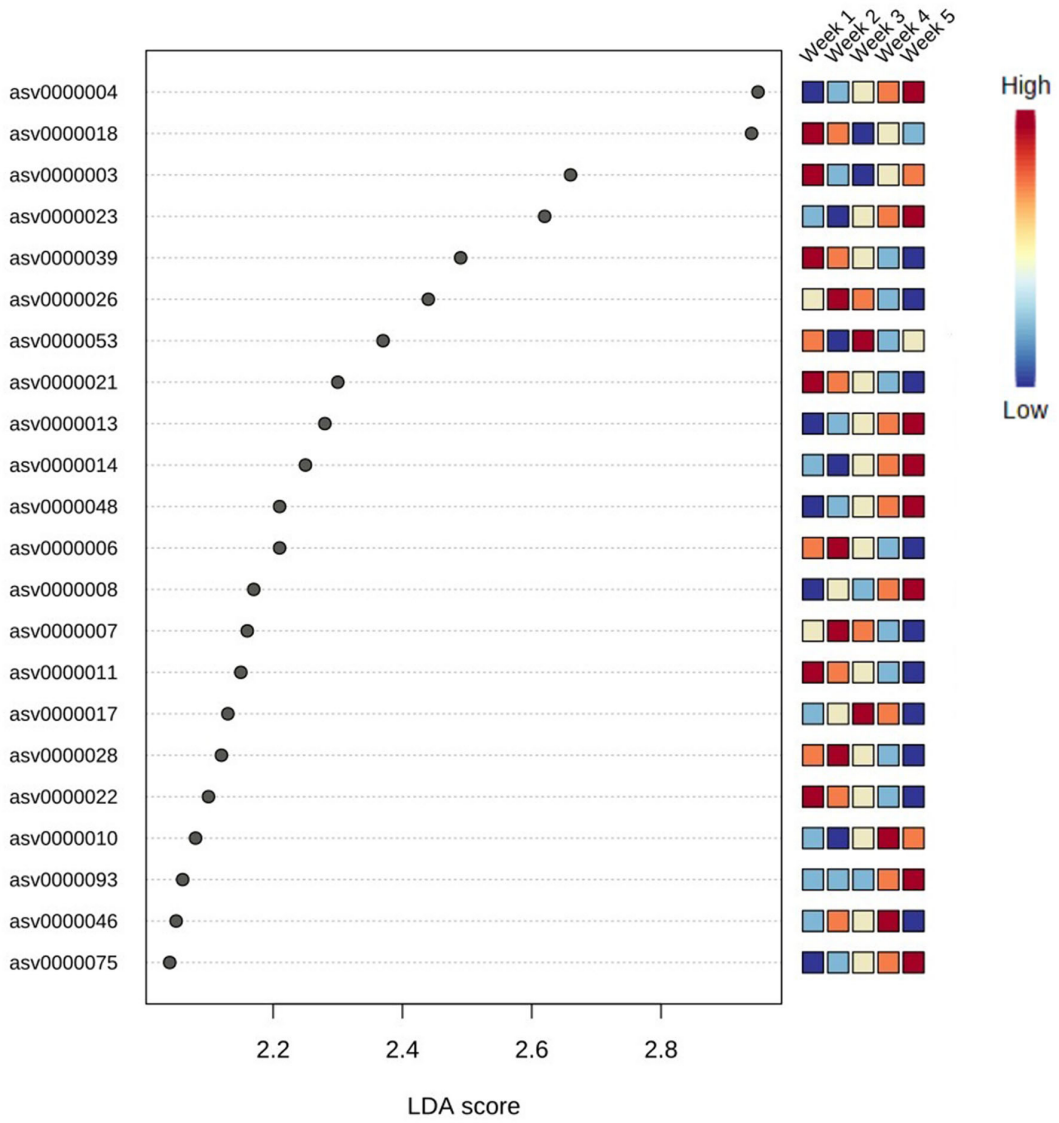
Top 22 bacteria identified as biomarkers in different weeks via linear discriminant analysis (LDA); significant taxa were ranked in decreasing order and defined at *p*-value < 0.05 and LDA score > 2.0. The heatmap on the right side shows whether the abundance of the taxa is high (red) or low (blue).

Differences in microbial assemblies along the temporal succession also resulted in 22 taxa that were identified as time biomarkers via the linear discriminant analysis (LDA) with *p*-value < 0.05 and LDA score > 2.0. In this study, time biomarkers are ASVs whose relative abundances differ significantly between samples that come from different timepoints. The highest number of biomarking microbes (8 features) belonged to the last week of community development, followed by the first week (6 features) and second week (4 features), while weeks 3 and 4 shared the same number of biomarkers (2 features) (Figure 6). Despite temporal dynamics, the following families always played an important part as biomarkers: *Saprospiraceae, Thiohalorhabdaceae, Sphingomonadaceae*, and *Rhodobacteraceae* (Table 1).

**Table 1:** List of biomarkers identified during a 5-week period. Bacteria were annotated at the lowest level of the genus.

**ASV No**.	**Week**	**Phylum**	**Class**	**Order**	**Family**	**Genus**
asv0000018	1	*Deinococcus-Thermus*	*Deinococci*	*Deinococcales*	*Trueperaceae*	*Truepera*
asv0000003	1	*Planctomycetes*	*Planctomycetacia*	*Pirellulales*	*Pirellulaceae*	*Blastopirellula*
asv0000039	1	*Planctomycetes*	*Planctomycetacia*	*Pirellulales*	*Pirellulaceae*	*Blastopirellula*
asv0000021	1	*Proteobacteria*	*Alphaproteobacteria*	*Caulobacterales*	*Hyphomonadaceae*	*Litorimonas*
asv0000011	1	*Bacteroidetes*	*Bacteroidia*	*Flavobacteriales*	*Flavobacteriaceae*	*Hyunsoonleella*
asv0000022	1	*Bacteroidetes*	*Bacteroidia*	*Chitinophagales*	*Saprospiraceae*	*Lewinella*
asv0000026	2	*Bacteroidetes*	*Bacteroidia*	*Chitinophagales*	*Saprospiraceae*	
asv0000006	2	*Bacteroidetes*	*Bacteroidia*	*Chitinophagales*	*Saprospiraceae*	*Portibacter*
asv0000007	2	*Planctomycetes*	*Planctomycetacia*	*Pirellulales*	*Pirellulaceae*	*Blastopirellula*
asv0000028	2	*Patescibacteria*	*Gracilibacteria*	*JGI_0000069-P22*		
asv0000053	3	*Bacteroidetes*	*Bacteroidia*	*Chitinophagales*	*Saprospiraceae*	
asv0000017	3	*Bacteroidetes*	*Bacteroidia*	*Chitinophagales*	*Saprospiraceae*	
asv0000010	4	*Proteobacteria*	*Alphaproteobacteria*	*Sphingomonadales*	*Sphingomonadaceae*	*Erythrobacter*
asv0000046	4	*Planctomycetes*	*Planctomycetacia*	*Planctomycetales*		
asv0000004	5	*Proteobacteria*	*Gammaproteobacteria*	*Thiohalorhabdales*	*Thiohalorhabdaceae*	*Granulosicoccus*
asv0000023	5	*Proteobacteria*	*Gammaproteobacteria*	*Thiohalorhabdales*	*Thiohalorhabdaceae*	*Granulosicoccus*
asv0000013	5	*Proteobacteria*	*Alphaproteobacteria*	*Sphingomonadales*	*Sphingomonadaceae*	*Erythrobacter*
asv0000014	5	*Proteobacteria*	*Alphaproteobacteria*	*Sphingomonadales*	*Sphingomonadaceae*	*Erythrobacter*
asv0000048	5	*Bacteroidetes*	*Bacteroidia*	*Chitinophagales*	*Saprospiraceae*	*Rubidimonas*
asv0000008	5	*Proteobacteria*	*Alphaproteobacteria*	*Caulobacterales*	*Hyphomonadaceae*	
asv0000093	5	*Proteobacteria*	*Alphaproteobacteria*	*Rhodobacterales*	*Rhodobacteraceae*	
asv0000075	5	*Proteobacteria*	*Alphaproteobacteria*	*Rhodobacterales*	*Rhodobacteraceae*	

## 4. Discussion

Macroalgae are known to form an inseparable relationship with their associated microbiome, which can possibly impact the seaweed's functioning and ecological services, given different conditions (Ren et al., 2022). To better understand the effect of temporal force on microbial assembly in macroalgae-associated microbiota, our study characterized the succession of an *Ulva fasciata*-associated microbial community from an IMTA biofilter system across 5 weeks of cultivation. Ecological indices were utilized to provide insights into the temporal changes of the microbiota, with a focus on biomarker species at each timepoint.

Previous studies indicated that IMTA conditions substantially influenced the structure and composition of *Ulva*-associated microbiota (Califano et al., 2020; Wichard, 2022). The most abundant phyla found in our study, such as *Proteobacteria, Bacteroidetes*, and *Planctomycetes*, were in strong agreement with other studies across various locations, especially in ITMA setups (Califano et al., 2020; Wiegand et al., 2021). *Proteobacteria* and *Bacteroidetes* were classified as two major groups of primary colonizers as many microbes belonging to these phyla are known to have a positive effect on seaweed growth and development (Wiegand et al., 2021; Ren et al., 2022). On the other hand, *Planctomycetes* played an essential role in the biofilm complex in a wide range of macroalgae due to their antibiotic resistance and dependence on sulfated polysaccharides supplied by algae (Wiegand et al., 2021). However, *Deinococcus-Thermus* has not been detected before in macroalgae from IMTA systems, although species in this taxon are resistant to extreme environments such as radioactive or thermophilic conditions (Ho et al., 2016). At the class level, our results agree with the prior study in the context of IMTA, where *Bacteroidia, Alpha- and Gamma- proteobacteria*, and *Planctomycetacia* were reported as among the most abundant taxa associated with macroalgae *Ulva* (Califano et al., 2020). At the family level, although *Saprospiraceae* was generally noticed to be prevalent in planktonic water and *Ulva* microbiome (Burke et al., 2011; Califano et al., 2020), it was characterized by a very low abundance of 4% in the aquaculture environment (Califano et al., 2020). In our study, however, this family was present in cultivated *Ulva* with the highest relative abundance of almost 20%.

Moreover, we also discovered other predominant families such as *Thiohalorhabdaceae* and *Pirellulaceae* which were not reported in other research studies about macroalgae originating from an IMTA environment. Being ubiquitous in hypersaline habitats, species belonging to the *Thiohalorhabdaceae* family (*Proteobacteria* phylum) can survive various extreme conditions due to their metabolic diversity, especially by sulfur-oxidizing using oxygen or nitrate as an electron acceptor (Sorokin and Merkel, 2023). Their success in the *Ulva* microbiome in our IMTA facility could be partly explained due to the high salinity of the Gulf of Aqaba ambient seawater, which is approximately 41% during winter-spring time (Al-Taani et al., 2020). While *Pirellulaceae* (*Planctomycetes* phylum) may appear in a broad range of brackish and marine habitats (Lage et al., 2015), their high abundance in marine macroalgae is suggested to be due to their ability to degrade sulfated polysaccharides produced by seaweeds and microalgae (Bengtsson and Øvreås, 2010).

We further identified the time biomarkers with potential properties and functions that encouraged their survival and high prevalence in *Ulva* microbiota. Species from the genus *Blastopirellula* (dominating phylum *Planctomycetes*), which were biomarkers in the first and second weeks, are popularly identified as macroalgae-associated taxa, notwithstanding geographical location (Lage and Bondoso, 2014; Califano et al., 2020; Wiegand et al., 2021). In week 4, the genus *Erythrobacter* (*Alphaproteobacteria* class), which is important and widespread in the ocean environment, was found to be a biomarker. The presence of extracellular quorum sensing signals in a cultivated candidate of this genus was believed to support the biofilm formation, thereby supporting their survival on the surface of macroalgae (Abdul Malik et al., 2020). In the last week, a biomarker of the genus *Granulosicoccus*, found in many marine regions and especially associated with diverse marine macroalgae, appeared. Full genome sequencing of a *Granulosicoccus* strain revealed certain genes responsible for the lyase activity of algae polysaccharides which could support their existence in algae (Kang et al., 2018).

Regarding microbial community dynamics, swift changes were found to occur in the *U. fasciata*-associated microbiota structure. This aspect of the microbial community in algae cultivated from an IMTA system has not been discussed previously. The intrinsic dynamic property of the bacterial community encourages new insight into microbial development in which a rapid change in the microbiota can facilitate better understanding and bioengineering of green macroalgae. The dynamics of microbial composition during a short period of time suggest that a finer time resolution should be considered when studying *Ulva*-associated microbiota in the context of an IMTA system, where considerably swift changes along its coastal area could be a crucial factor in determining community assembly.

## 5. Conclusion

The current study contributed to knowledge about the microbial community associated with the macroalgae *Ulva fasciata* cultivated in an IMTA system in the Gulf of Aqaba (Eilat, Israel). The *Ulva*-associated microbiome exhibited high dynamics in its composition and structure along the temporal succession, with the grouping of distinct communities at each timepoint. We suggest that future research should cover longer time series within various conditions and integrate the relationship between nutrient availability and microbial assembly to fully understand the ecological patterns and underlying forces governing the microbiota. Our findings of underestimated taxa (phylum *Deinococcus-Thermus*, families *Saprospiraceae, Thiohalorhabdaceae*, and *Pirellulaceae*) associated with *Ulva* in IMTA-specific conditions suggest that more attention should be given to this microbiota in such a unique habitat and that common technical issues in omics studies (the choice of primers, sequencing techniques, and bioinformatics workflows) should be taken into account during data mining and interpretation.

## Data availability statement

The dataset presented in this study can be found in the ENA online repository under the accession number PRJEB62134. Supplementary data and documents are provided in the article/Supplementary material.

## Author contributions

LG, OO, and DN: conceptualization and writing. DN and LG: investigation, interpretation of results, visualization, and editing. LG: funding acquisition, project administration, and supervision. All authors read and approved the final manuscript.
