# Supplementary material for: Temporal force governs the microbial assembly associated with Ulva fasciata (Chlorophyta) from an integrated multi-trophic aquaculture system
Source: Front Microbiol. 2023 Oct 5;14:1223204. doi: 10.3389/fmicb.2023.1223204 (PMC10585273; doi:10.3389/fmicb.2023.1223204)
Supplement: Supplementary file 1 [file Data_Sheet_1.docx]

Supplementary Material

Temporal force governs the microbial assembly associated with *Ulva fasciata* (Chlorophyta) from an integrated multi-trophic aquaculture system

Dzung Nguyen^1,2^, Ofer Ovadia^3^, and Lior Guttman^2,3*^

*** Correspondence:** Lior Guttman: [lior.guttman@mail.huji.ac.il](mailto:lior.guttman@ocean.org.il)

# Supplementary Figures


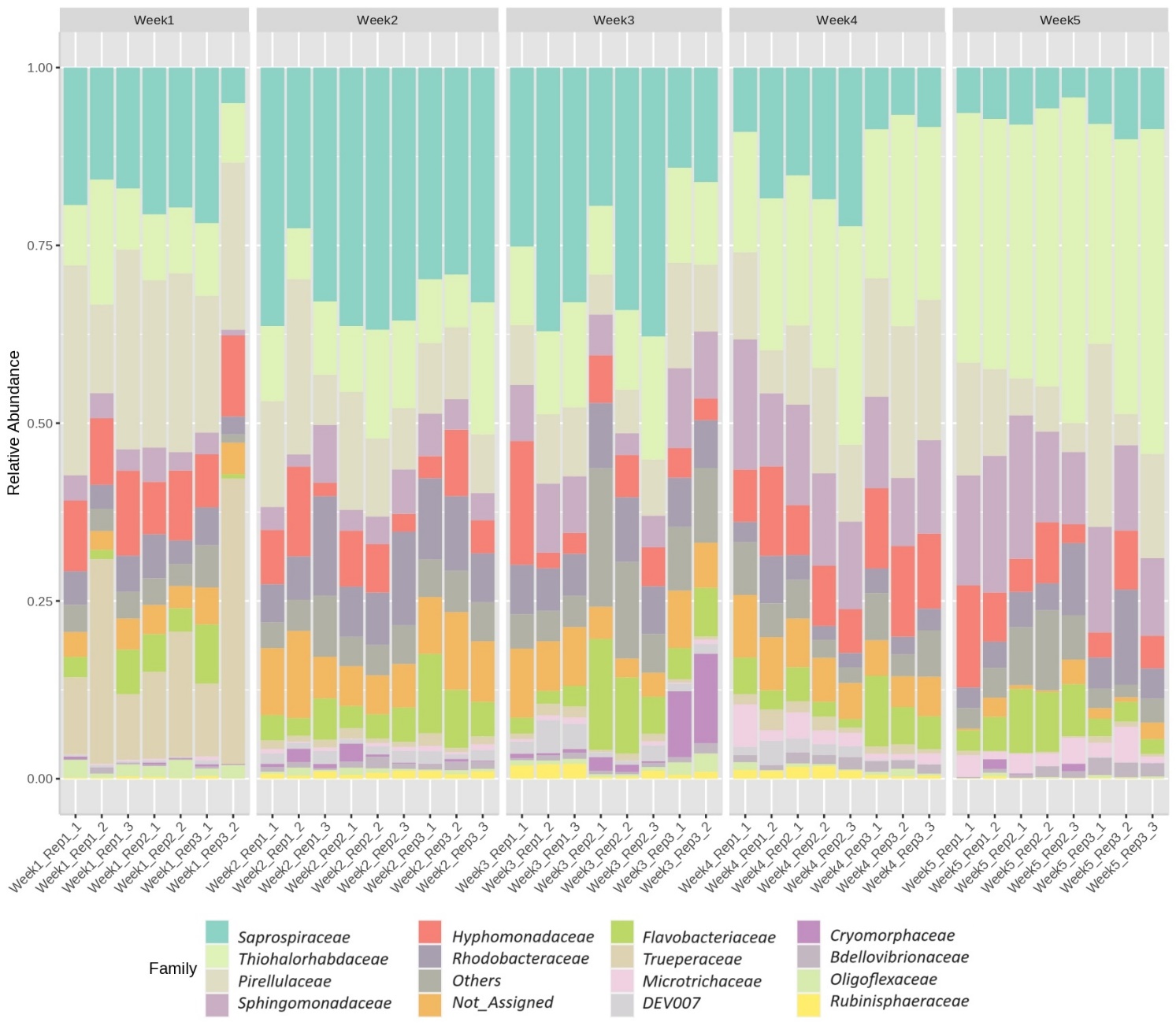


**Figure S1:** Taxonomic composition performed as relative abundance of the prokaryotes at the family level. List of the remaining families (“Others”) can be found in Table S5


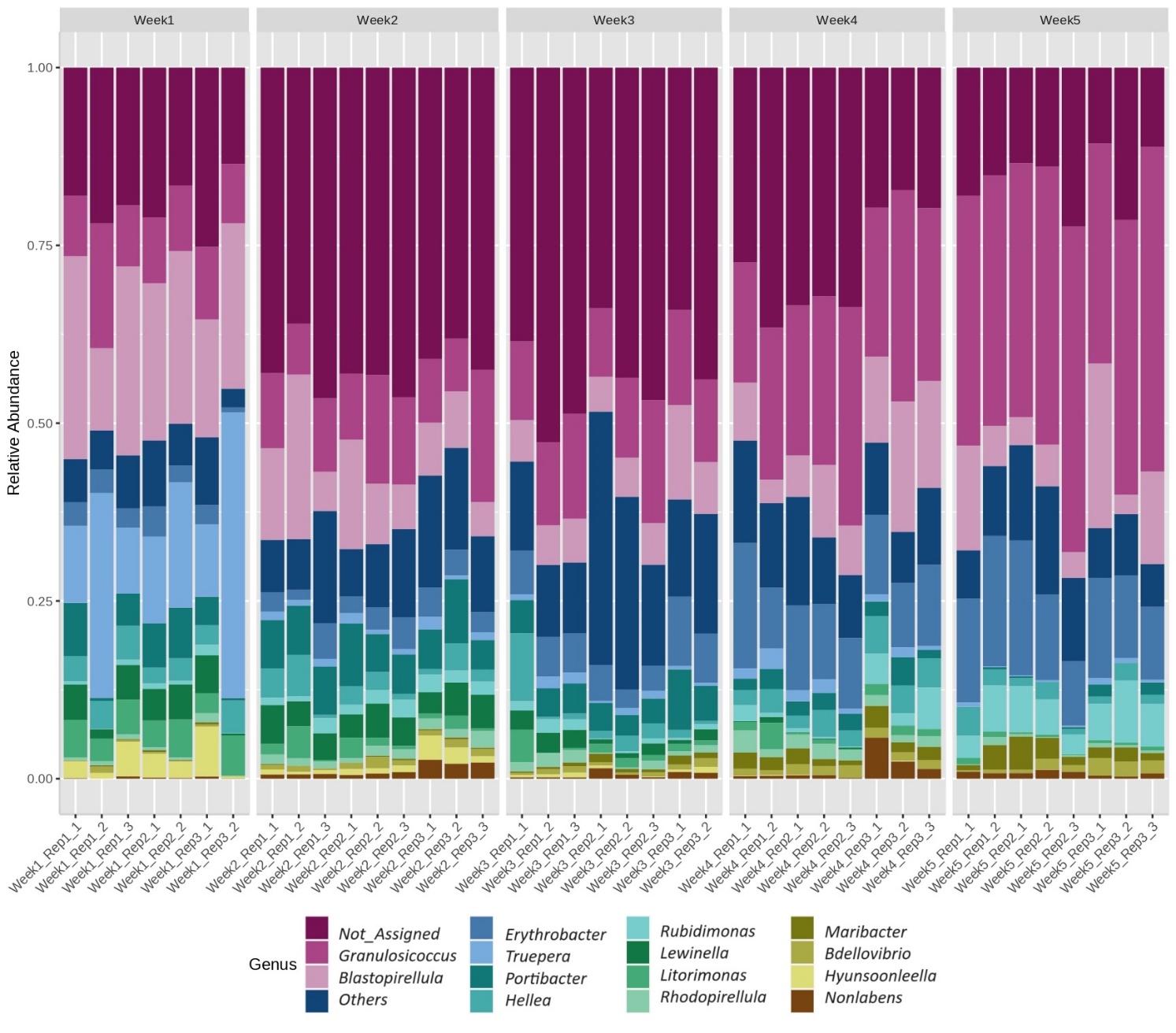


**Figure S2:** Taxonomic composition performed as relative abundance of the prokaryotes at the genus level. List of the remaining genus (“Others”) can be found in Table S6

# Supplementary Tables

**Table S1A:** Chemical analysis (TAN, NO3, PO4) in the inlet and outlet water of cultivated *U. fasciata* during five weeks

| **Date** | **# Tank** | **TAN/N mg/l** | **NO3/N mg/l** | **PO4/P mg/l** |
| --- | --- | --- | --- | --- |
| 5/3/2020 | Ulva1-inlet | 1.69 | 1.57 | 0.33 |
| 5/3/2020 | Ulva1-outlet | 0.57 | 0.64 | 0.20 |
| 5/3/2020 | Ulva2-inlet | 1.64 | 1.52 | 0.29 |
| 5/3/2020 | Ulva2-outlet | 0.77 | 0.73 | 0.16 |
| 5/3/2020 | Ulva3-inlet | 1.64 | 1.53 | 0.31 |
| 5/3/2020 | Ulva3-outlet | 0.68 | 0.65 | 0.16 |
| 9/3/2020 | Ulva1-inlet | 0.74 | 0.38 | 0.10 |
| 9/3/2020 | Ulva1-outlet | 0.21 | 0.45 | 0.04 |
| 9/3/2020 | Ulva2-inlet | 0.77 | 0.40 | 0.06 |
| 9/3/2020 | Ulva2-outlet | 0.31 | 0.47 | 0.12 |
| 9/3/2020 | Ulva3-inlet | 0.75 | 0.42 | 0.14 |
| 9/3/2020 | Ulva3-outlet | 0.38 | 0.42 | 0.14 |
| 11/3/2020 | Ulva1-inlet | 0.90 | 0.52 | 0.12 |
| 11/3/2020 | Ulva1-outlet | 0.79 | 0.83 | 0.14 |
| 11/3/2020 | Ulva2-inlet | 0.92 | 0.56 | 0.12 |
| 11/3/2020 | Ulva2-outlet | 0.80 | 0.92 | 0.10 |
| 11/3/2020 | Ulva3-inlet | 0.85 | 0.54 | 0.14 |
| 11/3/2020 | Ulva3-outlet | 0.48 | 0.69 | 0.12 |
| 18/3/2020 | Ulva1-inlet | 1.82 | 1.93 | 0.31 |
| 18/3/2020 | Ulva1-outlet | 1.36 | 1.92 | 0.26 |
| 18/3/2020 | Ulva2-inlet | 1.81 | 1.93 | 0.24 |
| 18/3/2020 | Ulva2-outlet | 1.37 | 2.03 | 0.31 |
| 18/3/2020 | Ulva3-inlet | 1.80 | 1.99 | 0.24 |
| 18/3/2020 | Ulva3-outlet | 1.35 | 2.04 | 0.39 |
| 26/3/2020 | Ulva1-inlet | 2.38 | 0.23 | 0.13 |
| 26/3/2020 | Ulva1-outlet | 2.09 | 0.71 | 0.17 |
| 26/3/2020 | Ulva2-inlet | 1.65 | 0.18 | 0.13 |
| 26/3/2020 | Ulva2-outlet | 1.83 | 0.00 | 0.07 |
| 26/3/2020 | Ulva3-inlet | 1.87 | 0.21 | 0.35 |
| 26/3/2020 | Ulva3-outlet | 1.15 | 0.11 | 0.25 |
| 2/4/2020 | Ulva1-inlet | 1.88 | 0.02 | 0.09 |
| 2/4/2020 | Ulva1-outlet | 2.24 | 0.29 | 0.15 |
| 2/4/2020 | Ulva2-inlet | 2.11 | 0.52 | 0.17 |
| 2/4/2020 | Ulva2-outlet | 1.61 | 0.14 | 0.03 |
| 2/4/2020 | Ulva3-inlet | 1.80 | 0.00 | 0.09 |
| 2/4/2020 | Ulva3-outlet | 2.50 | 1.68 | 0.57 |
| 3/4/2020 | Ulva1-inlet | 2.08 | 1.87 | 0.19 |
| 3/4/2020 | Ulva1-outlet | 1.55 | 2.16 | 0.03 |
| 3/4/2020 | Ulva2-inlet | 1.30 | 1.26 | 0.09 |
| 3/4/2020 | Ulva2-outlet | 2.84 | 0.35 | 0.05 |
| 3/4/2020 | Ulva3-inlet | 1.44 | 0.00 | 0.05 |
| 3/4/2020 | Ulva3-outlet | 1.56 | 0.30 | 0.13 |
| 9/4/2020 | Ulva1-inlet | 1.84 | 1.48 | 0.27 |
| 9/4/2020 | Ulva1-outlet | 0.85 | 1.58 | 0.29 |
| 9/4/2020 | Ulva2-inlet | 1.27 | 0.50 | 0.31 |
| 9/4/2020 | Ulva2-outlet | 1.56 | 1.71 | 0.21 |
| 9/4/2020 | Ulva3-inlet | 1.30 | 2.47 | 0.27 |
| 9/4/2020 | Ulva3-outlet | 0.86 | 0.23 | 0.21 |
|  | **Average Inlet** | **1.51** | **0.92** | **0.19** |
|  | **Standard Deviation Inlet** | **0.47** | **0.76** | **0.09** |
|  | **Average Outlet** | **1.24** | **0.88** | **0.18** |
|  | **Standard Deviation Outlet** | **0.70** | **0.69** | **0.12** |

**Table S1B:** Physical measurements (pH, dissolved oxygen, temperature) in the ambient water of cultivated *U. fasciata* during experimental period

| **Date** | **Tank** | **pH** | **O2 (%)** | **O2 (mg/l)** | **Temp (°C)** |
| --- | --- | --- | --- | --- | --- |
| 5/3/2020 | Ulva-Tank1 | 7.96 | 122 | 8.37 | 22 |
| 5/3/2020 | Ulva-Tank2 | 7.97 | 113.4 | 7.75 | 22.1 |
| 5/3/2020 | Ulva-Tank3 | 7.98 | 128.4 | 8.76 | 22.1 |
| 11/3/2020 | Ulva-Tank1 | 7.94 | 105.5 | 7.37 | 20.8 |
| 11/3/2020 | Ulva-Tank2 | 7.99 | 99.8 | 6.91 | 20 |
| 11/3/2020 | Ulva-Tank3 | 7.97 | 105.5 | 7.67 | 18.8 |
| 18/3/2020 | Ulva-Tank1 | 8.08 | 108.9 | 7.76 | 19.8 |
| 18/3/2020 | Ulva-Tank2 | 8.07 | 106 | 7.58 | 19.5 |
| 18/3/2020 | Ulva-Tank3 | 8.08 | 108.5 | 7.76 | 19.4 |
| 19/3/2020 | Ulva-Tank1 | - | 121.6 | 8.46 | 21.1 |
| 19/3/2020 | Ulva-Tank2 | - | 113.4 | 7.94 | 20.8 |
| 19/3/2020 | Ulva-Tank3 | - | 124.2 | 8.68 | 20.9 |
| 26/3/2020 | Ulva-Tank1 | 7.97 | 119 | 8.37 | 20.2 |
| 26/3/2020 | Ulva-Tank2 | 8.01 | 110.1 | 7.74 | 20.2 |
| 26/3/2020 | Ulva-Tank3 | 7.95 | 119.5 | 8.41 | 20.1 |
| 2/4/2020 | Ulva-Tank1 | - | 107.5 | 7.6 | 19.9 |
| 2/4/2020 | Ulva-Tank2 | - | 107.7 | 7.7 | 19.6 |
| 2/4/2020 | Ulva-Tank3 | - | 114 | 8.1 | 19.7 |
| 2/4/2020 | Ulva-Tank1 | 7.88 | 106.7 | 6.95 | 24.9 |
| 2/4/2020 | Ulva-Tank2 | 7.93 | 107.8 | 7.07 | 24.6 |
| 2/4/2020 | Ulva-Tank3 | 7.92 | 108 | 7.05 | 24.8 |
| 3/4/2020 | Ulva-Tank1 | 8.04 | 118.7 | 8.32 | 20.8 |
| 3/4/2020 | Ulva-Tank2 | 8.01 | 117.9 | 8.28 | 20.6 |
| 3/4/2020 | Ulva-Tank3 | 8.02 | 122.4 | 8.6 | 20.5 |
| 9/4/2020 | Ulva-Tank1 | 8.01 | 111.2 | 7.98 | 19.3 |
| 9/4/2020 | Ulva-Tank2 | 8.04 | 115.1 | 8.08 | 20.4 |
| 9/4/2020 | Ulva-Tank3 | 8.05 | 117.2 | 8.3 | 19.9 |
| **Mean** | | **7.99** | **113.33** | **7.91** | **20.84** |
| **Standard Deviation** | | **0.05** | **6.93** | **0.52** | **1.60** |

**Table S2A:** List of *Ulva* samples that were collected during the five-week experiment. Each sample name indicates biological replicate/tank (Rep1/2/3) and technical replicate (number 1/2/3 at the end)

| **Sample Name** | **Time** | **Tank** | **Technical replicate** | **Sample Name** | **Time** | **Tank** | **Technical replicate** |
| --- | --- | --- | --- | --- | --- | --- | --- |
| Week1_Rep1_1 | Week 1 | Tank 1 | 1 | Week3_Rep2_2 | Week 3 | Tank 2 | 2 |
| Week1_Rep1_2 | Week 1 | Tank 1 | 2 | Week3_Rep2_3 | Week 3 | Tank 2 | 3 |
| Week1_Rep1_3 | Week 1 | Tank 1 | 3 | Week3_Rep3_1 | Week 3 | Tank 3 | 1 |
| Week1_Rep2_1 | Week 1 | Tank 2 | 1 | Week3_Rep3_2 | Week 3 | Tank 3 | 2 |
| Week1_Rep2_2 | Week 1 | Tank 2 | 2 | Week4_Rep1_1 | Week 4 | Tank 1 | 1 |
| Week1_Rep3_1 | Week 1 | Tank 3 | 1 | Week4_Rep1_2 | Week 4 | Tank 1 | 2 |
| Week1_Rep3_2 | Week 1 | Tank 3 | 2 | Week4_Rep2_1 | Week 4 | Tank 2 | 1 |
| Week2_Rep1_1 | Week 2 | Tank 1 | 1 | Week4_Rep2_2 | Week 4 | Tank 2 | 2 |
| Week2_Rep1_2 | Week 2 | Tank 1 | 2 | Week4_Rep2_3 | Week 4 | Tank 2 | 3 |
| Week2_Rep1_3 | Week 2 | Tank 1 | 3 | Week4_Rep3_1 | Week 4 | Tank 3 | 1 |
| Week2_Rep2_1 | Week 2 | Tank 2 | 1 | Week4_Rep3_2 | Week 4 | Tank 3 | 2 |
| Week2_Rep2_2 | Week 2 | Tank 2 | 2 | Week4_Rep3_3 | Week 4 | Tank 3 | 3 |
| Week2_Rep2_3 | Week 2 | Tank 2 | 3 | Week5_Rep1_1 | Week 5 | Tank 1 | 1 |
| Week2_Rep3_1 | Week 2 | Tank 3 | 1 | Week5_Rep1_2 | Week 5 | Tank 1 | 2 |
| Week2_Rep3_2 | Week 2 | Tank 3 | 2 | Week5_Rep2_1 | Week 5 | Tank 2 | 1 |
| Week2_Rep3_3 | Week 2 | Tank 3 | 3 | Week5_Rep2_2 | Week 5 | Tank 2 | 2 |
| Week3_Rep1_1 | Week 3 | Tank 1 | 1 | Week5_Rep2_3 | Week 5 | Tank 2 | 3 |
| Week3_Rep1_2 | Week 3 | Tank 1 | 2 | Week5_Rep3_1 | Week 5 | Tank 3 | 1 |
| Week3_Rep1_3 | Week 3 | Tank 1 | 3 | Week5_Rep3_2 | Week 5 | Tank 3 | 2 |
| Week3_Rep2_1 | Week 3 | Tank 2 | 1 | Week5_Rep3_3 | Week 5 | Tank 3 | 3 |

**Table S2B:** List of original, filtered, and retained sequence counts in each sample and the retained percentage (%) after removing Chloroplast and Mitochondria.

| **Sample** | **Original** | **Filtered** | **Retained** | **Percent Retained (%)** |
| --- | --- | --- | --- | --- |
| Week1_Rep1_1 | 35873 | 7070 | 28803 | 80.29 |
| Week1_Rep1_2 | 56700 | 40451 | 16249 | 28.66 |
| Week1_Rep1_3 | 54798 | 19653 | 35145 | 64.14 |
| Week1_Rep2_1 | 50634 | 14565 | 36069 | 71.23 |
| Week1_Rep2_2 | 48578 | 10008 | 38570 | 79.4 |
| Week1_Rep3_1 | 59392 | 24320 | 35072 | 59.05 |
| Week1_Rep3_2 | 58231 | 47579 | 10652 | 18.29 |
| Week2_Rep1_1 | 85325 | 33531 | 51794 | 60.7 |
| Week2_Rep1_2 | 57509 | 20993 | 36516 | 63.5 |
| Week2_Rep1_3 | 22616 | 4412 | 18204 | 80.49 |
| Week2_Rep2_1 | 40048 | 18461 | 21587 | 53.9 |
| Week2_Rep2_2 | 81299 | 34676 | 46623 | 57.35 |
| Week2_Rep2_3 | 41016 | 14697 | 26319 | 64.17 |
| Week2_Rep3_1 | 57439 | 14788 | 42651 | 74.25 |
| Week2_Rep3_2 | 69133 | 25850 | 43283 | 62.61 |
| Week2_Rep3_3 | 97026 | 30057 | 66969 | 69.02 |
| Week3_Rep1_1 | 59376 | 44902 | 14474 | 24.38 |
| Week3_Rep1_2 | 48912 | 21321 | 27591 | 56.41 |
| Week3_Rep1_3 | 86854 | 42855 | 43999 | 50.66 |
| Week3_Rep2_1 | 60780 | 49100 | 11680 | 19.22 |
| Week3_Rep2_2 | 53941 | 42710 | 11231 | 20.82 |
| Week3_Rep2_3 | 67496 | 48959 | 18537 | 27.46 |
| Week3_Rep3_1 | 93215 | 54929 | 38286 | 41.07 |
| Week3_Rep3_2 | 90364 | 45975 | 44389 | 49.12 |
| Week4_Rep1_1 | 60928 | 14878 | 46050 | 75.58 |
| Week4_Rep1_2 | 40070 | 9702 | 30368 | 75.79 |
| Week4_Rep2_1 | 71459 | 22798 | 48661 | 68.1 |
| Week4_Rep2_2 | 61832 | 28778 | 33054 | 53.46 |
| Week4_Rep2_3 | 58593 | 21520 | 37073 | 63.27 |
| Week4_Rep3_1 | 80851 | 36450 | 44401 | 54.92 |
| Week4_Rep3_2 | 86299 | 45639 | 40660 | 47.12 |
| Week4_Rep3_3 | 70439 | 32870 | 37569 | 53.34 |
| Week5_Rep1_1 | 58691 | 35744 | 22947 | 39.1 |
| Week5_Rep1_2 | 50504 | 15620 | 34884 | 69.07 |
| Week5_Rep2_1 | 58884 | 39966 | 18918 | 32.13 |
| Week5_Rep2_2 | 64812 | 46107 | 18705 | 28.86 |
| Week5_Rep2_3 | 62389 | 36269 | 26120 | 41.87 |
| Week5_Rep3_1 | 42880 | 11189 | 31691 | 73.91 |
| Week5_Rep3_2 | 37242 | 16980 | 20262 | 54.41 |
| Week5_Rep3_3 | 56271 | 22411 | 33860 | 60.17 |
| **Average** | | | | **53.98** |

**Table S3**: List of all prokaryotic phyla found in the *Ulva* associated microbiota (in %)

| **Phylum** | **Percentage** |
| --- | --- |
| *Proteobacteria* | 48.26797707 |
| *Bacteroidetes* | 27.59599719 |
| *Planctomycetes* | 15.52913022 |
| *Deinococcus_Thermus* | 4.015838263 |
| *Patescibacteria* | 1.755703813 |
| *Actinobacteria* | 1.205438992 |
| *Verrucomicrobia* | 1.104858425 |
| *Cyanobacteria* | 0.226624568 |
| Not_Assigned | 0.185883072 |
| *Chloroflexi* | 0.103890813 |
| *Nanoarchaeaeota* | 0.008657567 |

**Table S4**: List of all classes found in the *Ulva*-associated microbiota (in %)

| **Class** | **Week_1** | **Week_2** | **Week_3** | **Week_4** | **Week_5** | **Total** |
| --- | --- | --- | --- | --- | --- | --- |
| *Bacteroidia* | 22.879 | 40.416 | 38.260 | 19.723 | 14.501 | 27.156 |
| *Alphaproteobacteria* | 17.293 | 21.138 | 23.538 | 26.765 | 27.474 | 23.242 |
| *Gammaproteobacteria* | 11.613 | 14.445 | 18.958 | 27.717 | 41.031 | 22.753 |
| *Planctomycetacia* | 23.834 | 15.003 | 11.114 | 17.346 | 11.149 | 15.689 |
| *Deinococci* | 18.569 | 1.034 | 0.890 | 1.390 | 0.387 | 4.454 |
| *Deltaproteobacteria* | 2.736 | 2.011 | 2.256 | 2.042 | 1.580 | 2.125 |
| *Gracilibacteria* | 2.162 | 3.222 | 1.753 | 0.969 | 0.370 | 1.695 |
| *Acidimicrobiia* | 0.207 | 0.619 | 0.448 | 2.224 | 2.478 | 1.195 |
| *Verrucomicrobiae* | 0.281 | 1.480 | 2.199 | 1.336 | 0.079 | 1.075 |
| *Oxyphotobacteria* | 0.009 | 0.005 | 0.067 | 0.177 | 0.791 | 0.210 |
| Not_Assigned | 0.290 | 0.300 | 0.253 | 0.093 | 0.023 | 0.192 |
| *Anaerolineae* | 0.057 | 0.158 | 0.073 | 0.149 | 0.070 | 0.101 |
| *OM190* | 0.064 | 0.124 | 0.052 | 0.019 | 0.037 | 0.059 |
| *Parcubacteria* | 0.006 | 0.035 | 0.047 | 0.050 | 0.028 | 0.033 |
| *Melainabacteria* | 0.000 | 0.008 | 0.042 | 0.001 | 0.003 | 0.011 |
| *Woesearchaeia* | 0.001 | 0.001 | 0.041 | 0.000 | 0.000 | 0.009 |
| Others | 0.000 | 0.000 | 0.009 | 0.000 | 0.000 | 0.002 |

**Table S5**: List of all prokaryotic families found in the *Ulva*-associated microbiota (in %)

| **Family** | **Percentage** | **Family** | **Percentage** |
| --- | --- | --- | --- |
| *Saprospiraceae* | 19.77185 | *Vibrionaceae* | 0.120951 |
| *Thiohalorhabdaceae* | 19.32089 | *NS9_marine_group* | 0.115349 |
| *Pirellulaceae* | 13.59773 | *Methylophagaceae* | 0.110511 |
| *Sphingomonadaceae* | 8.459462 | *Rubritaleaceae* | 0.107456 |
| *Hyphomonadaceae* | 7.558566 | *Synechococcales_Incertae_Sedis* | 0.09065 |
| *Rhodobacteraceae* | 6.126757 | *Cyclobacteriaceae* | 0.088358 |
| *Not_Assigned* | 5.258708 | *Woeseiaceae* | 0.087849 |
| *Flavobacteriaceae* | 4.991597 | *Xenococcaceae* | 0.080464 |
| *Trueperaceae* | 4.015838 | *Haliangiaceae* | 0.065186 |
| *Microtrichaceae* | 1.176665 | *Saccharospirillaceae* | 0.064422 |
| *DEV007* | 0.997403 | *Colwelliaceae* | 0.054237 |
| *Cryomorphaceae* | 0.915665 | *Caldilineaceae* | 0.051945 |
| *Bdellovibrionaceae* | 0.914137 | *Cellvibrionaceae* | 0.050927 |
| *Oligoflexaceae* | 0.739458 | *Thiotrichaceae* | 0.044306 |
| *Alteromonadaceae* | 0.700499 | *Halieaceae* | 0.034121 |
| *Rubinisphaeraceae* | 0.671725 | *Sedimenticolaceae* | 0.02419 |
| *Spirosomaceae* | 0.546445 | *Sandaracinaceae* | 0.018843 |
| *Burkholderiaceae* | 0.538806 | *Rickettsiaceae* | 0.018079 |
| *Schleiferiaceae* | 0.497046 | *Terasakiellaceae* | 0.016042 |
| *Bacteriovoracaceae* | 0.361581 | *Porticoccaceae* | 0.014769 |
| *Crocinitomicaceae* | 0.293339 | *SM2D12* | 0.014769 |
| *Nitrincolaceae* | 0.257945 | *Methylophilaceae* | 0.014005 |
| *Rhizobiaceae* | 0.257945 | *Methyloligellaceae* | 0.013496 |
| *NS11_12_marine_group* | 0.217967 | *Unknown_Family* | 0.008403 |
| *Pseudoalteromonadaceae* | 0.186392 | *Acaryochloridaceae* | 0.003056 |
| *Ectothiorhodospiraceae* | 0.16704 | *Others* | 0.002801 |
| *Micavibrionaceae* | 0.140813 | *Spongiibacteraceae* | 0.002546 |

**Table S6**: List of all prokaryotic genera found in the *Ulva*-associated microbiota (in %)

| **Genus** | **Percentage** | **Genus** | **Percentage** |
| --- | --- | --- | --- |
| Not_Assigned | 30.1413215 | *Roseibacillus* | 0.074608 |
| *Granulosicoccus* | 19.32089025 | *Ruegeria* | 0.072062 |
| *Blastopirellula* | 11.2339581 | *Loktanella* | 0.071298 |
| *Erythrobacter* | 7.281778373 | *NS3a_marine_group* | 0.06977 |
| *Truepera* | 4.015838257 | *Haliangium* | 0.065186 |
| *Portibacter* | 3.687359953 | *Thalassobius* | 0.063149 |
| *Hellea* | 2.675188433 | *Nitratireductor* | 0.057293 |
| *Rubidimonas* | 2.113974332 | *Litoribrevibacter* | 0.055001 |
| *Lewinella* | 1.959411285 | *Thalassotalea* | 0.054237 |
| *Litorimonas* | 1.692809126 | *Marine_Methylotrophic_Group_3* | 0.053728 |
| *Rhodopirellula* | 1.00911591 | *Marinagarivorans* | 0.04558 |
| *Maribacter* | 0.984671012 | *Aureispira* | 0.042524 |
| *Bdellovibrio* | 0.90013241 | *Thiothrix* | 0.036413 |
| *Hyunsoonleella* | 0.882053371 | *Sulfitobacter* | 0.03463 |
| *Nonlabens* | 0.851751885 | *Congregibacter* | 0.034121 |
| *Litoreibacter* | 0.847677735 | *Algicola* | 0.033102 |
| *Rubripirellula* | 0.821195763 | *Lutibacter* | 0.032339 |
| *Sva0996_marine_group* | 0.809227949 | *Rubritalea* | 0.032339 |
| *Hyphomonas* | 0.599663888 | *Pseudooctadecabacter* | 0.029283 |
| *Taeseokella* | 0.546445305 | *Poseidonocella* | 0.0247 |
| *Pirellula* | 0.527347729 | *Marinovum* | 0.023936 |
| *Lautropia* | 0.524546745 | *Hoppeia* | 0.021135 |
| *Schleiferia* | 0.497046241 | *Silicimonas* | 0.019352 |
| *Aliiroseovarius* | 0.477948666 | *Paraglaciecola* | 0.019352 |
| *Fuerstia* | 0.406396415 | *Lacinutrix* | 0.018334 |
| *Glaciecola* | 0.365400285 | *Nereida* | 0.016806 |
| *Alteromonas* | 0.315237319 | *Aestuariispira* | 0.016042 |
| *Aquimarina* | 0.301741699 | *Altererythrobacter* | 0.016042 |
| *Salinirepens* | 0.282898757 | *SAR92_clade* | 0.014769 |
| *Winogradskyella* | 0.228916277 | *Aquibacter* | 0.014769 |
| *Peredibacter* | 0.22153188 | *Pseudodonghicola* | 0.01426 |
| *Lentilitoribacter* | 0.200651865 | *Croceitalea* | 0.014005 |
| *Polaribacter_4* | 0.196832349 | *OM27_clade* | 0.014005 |
| *Roseobacter* | 0.195049908 | *Methylotenera* | 0.014005 |
| *Robiginitomaculum* | 0.191739662 | *Psychrosphaera* | 0.011968 |
| *Tenacibaculum* | 0.164239153 | *Fluviicola* | 0.009931 |
| *Pseudophaeobacter* | 0.155072316 | *Oleispira* | 0.009421 |
| *Pseudoalteromonas* | 0.141322061 | *Marinicella* | 0.008403 |
| *Dokdonia* | 0.133683032 | *Fretibacter* | 0.008403 |
| *Vibrio* | 0.120951314 | *WDS1C4* | 0.008148 |
| *Halobacteriovorax* | 0.11662253 | *Candidatus_Navis* | 0.007894 |
| *Lentibacter* | 0.114585455 | *Pir4_lineage* | 0.006111 |
| *Aurantivirga* | 0.090649827 | *Marinobacterium* | 0.005347 |
| *Schizothrix_LEGE_07164* | 0.090649827 | *Cellulophaga* | 0.004583 |
| *Woeseia* | 0.087848849 | Others | 0.004583 |
| *Muricauda* | 0.087084946 | *Acaryochloris_MBIC11017* | 0.003056 |
| *Xenococcus_PCC_7305* | 0.080464453 | *Donghicola* | 0.003056 |
